# Serum levels of 25‐hydroxy‐vitamin D in patients with oral squamous cell carcinoma: Making a case for chemoprevention

**DOI:** 10.1002/cre2.294

**Published:** 2020-04-04

**Authors:** Samuel E. Udeabor, Abdullah M. Albejadi, Waleed A. K. Al‐Shehri, Chidozie I. Onwuka, Saeed Y. Al‐Fathani, Abdullah A. Al Nazeh, Saleh F. Aldhahri, Faleh A. Alshahrani

**Affiliations:** ^1^ Oral and Maxillofacial Surgery Department, College of Dentistry King Khalid University Abha Saudi Arabia; ^2^ Department of Pediatrics and Orthodontics King Khalid University Abha Saudi Arabia; ^3^ College of Medicine King Saud University and King Fahad Medical City Riyadh Saudi Arabia; ^4^ Oral and Maxillofacial Surgery Department King Fahad Medical City Riyadh Saudi Arabia

**Keywords:** 25‐Hydroxy‐vitamin D, Oral squamous cell carcinoma, Chemoprevention

## Abstract

**Objectives:**

Serum level of vitamin D has been used as a predictor for cancer development. We intend to measure the baseline vitamin D level in patients with oral squamous cell carcinoma (OSCC) and to compare same with non‐cancer controls to determine any association.

**Materials and methods:**

Patients with OSCC presenting to our clinics were included in this study. Their baseline serum vitamin D levels were measured prior to cancer treatment after obtaining their consents. These patients were then matched with at least 2 cancer‐free subjects to serve as controls and whose serum vitamin D levels were also measured. The serum vitamin D levels obtained for the two groups were then categorized into **normal** (>35 ng/ml), **mild deficiency** (25–35 ng/ml), **moderate deficiency** (12.5–25 ng/ml), and **severe deficiency** (<12.5 ng/ml). The data were analyzed statistically and the two groups compared.

**Results:**

A total of 51 patients with OSCC (Male 22 [43%] and female 29 [57%]) and 113 cancer‐free controls (Male 36 [31.86%] and female 77 [68.14%]) were included in the study. The commonest site for OSCC was the tongue, accounting for 45% of the cancer cases. Mean age for cancer patients was 59.33 years ±12.54 and 49.24 years ±15.79 for the control. Among the OSCC patients, 74.51% had moderate to severe vitamin D deficiencies, whereas only 20.35% had a moderate deficiency in the control group with no severe deficiency.

**Conclusion:**

Logistic regression analysis shows a positive association between vitamin D deficiency and OSCC risk especially in levels below 25 ng/ml. This further corroborates the assertion that vitamin D deficiency may be a useful indicator of OSCC. It may, therefore, be necessary to routinely prescribe vitamin D supplements to subjects with moderate to severe deficiencies in order to decrease the chances of OSCC development.

## 1 INTRODUCTION

Oral squamous cell carcinoma (OSCC), which is a component of head and neck squamous cell carcinoma (HNSCC), is a disease that afflicts mostly the elderly and rare in patients below 40 years of age (Udeabor, Rana, Wegener, Gellrich, & Eckardt, 2012). It accounts for about 95% of all cases of HNSCC (Warnakulasuriya, 2009). The incidence of OSCC is given to be between 2 and 4% of all cancer cases worldwide (Markopoulos, 2012), making it the sixth most common cancer globally (Coelho, 2012). However, there is a much higher incidence in some Asian countries like India and Pakistan where it is reported to be as high as 30 and 10%, respectively (Coelho, 2012; Markopoulos, 2012). In the Middle East including Saudi Arabia, it forms only 2% of all the cancer cases seen (Coelho, 2012).

Etiology of OSCC is indeed multifactorial and many risk factors have been implicated for its development. The two most important risk factors, especially in the western world, are alcohol consumption and tobacco use (Markopoulos, 2012). In other ethnic populations, betel quid and areca nut chewing and the use of narcotics and cannabis are also significant risk factors (Coelho, 2012; Igbal et al., 2014; Markopoulos, 2012). Additionally, many studies have shown that viral infections like Human Papilloma Virus (HPV), Ebstein‐Barr virus (EBV), Hepatitis C Virus (HCV), and Human Immunodeficiency Virus (HIV) may all have roles to play in the etiology of oral cancer (Chole, Patil, Basak, Palandurkar, & Bhowate, 2010; Igbal et al., 2014; Jalouli et al., 2010; Markopoulos, 2012; Nagao & Sata, 2009 and Zygogianni et al., 2011).

These extrinsic factors and a host of intrinsic factors lead to complex molecular and cellular changes in a multistep process that eventually evolves to oral cancer (Choudhari, Chaudhary, Gadbail, Sharma, & Tekade, 2014). In this complex process, a study (Sinha, Mukhopadhyay, Das, Panda, & Bhutia, 2013) identified resistance to apoptosis (programmed cell death) by a group of cells known as cancer stem cells as an important factor. This, they claim, confers a “protective autophagy” on these cancer cells and therefore prolong their lifespans. This is believed to be responsible for the cancer metastasis, recurrence, and resistance to both chemotherapy and radiotherapy (Grimm et al., 2015; Sinha et al., 2013). Therefore, treatment options that will promote apoptosis will indeed help in the management of OSCC (Grimm et al., 2015).

The active form of vitamin D (1‐alpha, 25‐dihydroxycholecalciferol) (VD) has been shown through several studies to affect normal and cancerous cells by enhancing anti‐proliferation and pro‐apoptotic factors, as well as inhibition of cell‐cycle promoters and growth factor signaling pathways (Grimm et al., 2015; Grimm, Alexander, Munz, Hoffmann, & Reinert, 2013; Osafi et al., 2014 and Russel, Rassnick, Erb, Vaughan, & McDonough, 2010). This means that it is pro‐apoptotic and is a useful anti‐neoplastic agent in the management of several malignancies including those of the head and neck region (Grimm et al., 2015; Osafi et al., 2014 and Russel et al., 2010). Serum level of vitamin D has also been used as a predictor for cancer development (Grimm et al., 2015; Tuohimaa & Lou, 2012).

We, therefore, aim to measure the serum vitamin D levels of OSCC patients visiting our Maxillofacial Centers and comparing it with those in normal healthy OSCC‐free patients to determine any associations. This may further elucidate the significance or importance of vitamin D in the etiology and prevention of OSCC.

## 2 MATERIALS AND METHODS

The ethical review committees of King Fahad Medical City (KFMC), Riyadh, and College of Dentistry and King Khalid University (KKU), Abha, approved this study. Consecutive patients attending these hospitals and who were histologically diagnosed with OSCC were included in this study. Demographic data were recorded for these patients and additional information regarding tumor site, duration, and treatment received if any were also documented. Their baseline serum vitamin D levels were measured prior to the cancer treatment after obtaining their consents. These patients were then matched with at least two cancer‐free subjects to serve as controls and whose serum vitamin D levels were also measured. Patients who have commenced any form of cancer treatment were excluded from this study. In addition, patients and controls that are on any form of vitamin D supplements were also excluded.

The serum vitamin D levels obtained for the two groups were then categorized into **normal** (>35 ng/ml), **mild deficiency** (25–35 ng/ml), **moderate deficiency** (12.5–25 ng/ml), and **severe deficiency** (<12.5 ng/ml) (Alipour et al., 2014). The data generated were analyzed statistically using SPSS Statistics package Version 20 software (IBM, Chicago, IL) and descriptive statistics applied. Chi‐square test of association was perfumed to assess the association between the two groups. Simple logistics regression analysis was applied to see the association between the levels of Vitamin D in cancer cases and controls. Statistical significance was set at 5% level of significance (*p* < .05).

## 3 RESULTS

A total of 51 patients with OSCC and 113 cancer‐free controls were included in the study. Among the cancer patients, 22 (43%) were male and 29 (57%) female; whereas 36 (31.9%) were male and 77 (68.1%) female among the cancer‐free controls. The commonest site for OSCC was the tongue, accounting for 45% of the cancer cases (Table 1). Mean age for cancer patients was 59.33 years ±12.54, whereas it was 49.24 years ±15.79 for the control. Table 2 gives a full analysis of age group analysis and comparison between the OSCC cases and controls. Only five patients were below the age of 40 years for the cancer group. Among the OSCC patients, 74.51% had moderate to severe vitamin D deficiencies, whereas only 20.35% had a moderate deficiency in the control group with none severely deficient (Table 3). Logistic regression analysis for moderate and severe deficiency gave an odds ratio (OR) of 1.65 with a confidence interval (CI) of 0.98–2.77 (Table 4). Furthermore, independent *t*‐test comparing the 2 groups in relation to vitamin D deficiency, revealed a mean of 20.42 ± 12.02 for the cancer group and 34.99 ± 12.38 for the control group with a *p*‐value of .001.

**TABLE 1:** Characteristics of OSCC patients

Tumor site	Tongue	Retro‐molar	Lip	Mandible	Floor of the mouth	Alveolar bone	Buccal mucosa	Palate	Other sites^a^
No (51)	23	4	2	6	2	4	5	1	4
% (100)	45.0	7.8	4.0	11.8	4.0	7.8	9.8	2.0	7.8

Age group (years)	<30	31–40	41–50	51–60	61–70	71–80	81–90	>90
No (51)	1	4	3	20	15	7	0	1
% (100)	2.0	7.8	5.9	39.2	29.4	13.7	0.0	2.0

a Tonsils and unattached gingiva.

**TABLE 2:** Comparison of control and cancer patients by age groups

Age groups	Control group	%	Cancer group	%	Total	%
<=30 yrs	10	8.85	1	1.96	11	6.71
31–39 yrs	30	26.55	4	7.84	34	20.73
40–49 yrs	25	22.12	3	5.88	28	17.07
50–59 yrs	19	16.81	21	41.18	40	24.39
60–69 yrs	21	18.58	14	27.45	35	21.34
>=70 yrs	8	7.08	8	15.69	16	9.76
Total	113	100.00	51	100.00	164	100.00

*Note:* Chi‐square = 26.3601, *p* < .001.

**TABLE 3:** Comparison of OSCC patients and control by prevalence of Vit‐D deficiency

Vit‐D deficiency categories	OSCC patients	%	Control group	%	Total	%
Normal level (>35 ng/ml)	8	15.69	53	46.90	61	37.20
Mild deficiency (25–35 ng/ml)	5	9.80	37	32.74	42	25.61
Moderate deficiency (12.5–25 ng/ml)	24	47.06	23	20.35	47	28.66
Severe deficiency (< 12.5 ng/ml)	14	27.45	0	0.00	14	8.54
Total	51	100.00	113	100.00	164	100.00

*Note:* Chi‐square = 43.7150, *p* < .001.

Abbreviation: OSCC, oral squamous cell carcinoma.

**TABLE 4:** Logistic regression analysis of cases by vitamin D level

Vit‐D deficiency	Control	Cancer	OR	95% CI for OR		*p*‐value
Normal level	53	8	Ref.			
Mild deficiency	37	5	0.14	0.05	0.34	.0001*
Moderate and severe deficiency	23	38	1.65	0.98	2.77	.0570

* *p* < .05.

Abbreviations: CI, confidence interval; OR, odds ratio.

## 4 DISCUSSION

The chemopreventive and therapeutic roles of vitamin D in a wide range of human cancers have been suggested in the literature (Bikle, Oda, & Teichert, 2011; Kamradt et al., 2003). Indeed, many of these malignant lesions including OSCC have been shown to express the receptor for 1,25 Dihydroxyvitamin D_3_; also known as VDR (vitamin D receptor) (Adisa et al., 2017; Grimm et al., 2013 and 2015; Osafi et al., 2014 and Russel et al., 2010). The suggested mechanism of action may be through its effect on p53‐mediated DNA damage in cases of skin squamous cell carcinoma (Reichrath & Reichrath, 2013). As for OSCC, Osafi et al. (2014) via an in vitro study opined that the activation of two apoptotic pathways (caspase and bcl:bax) by vitamin D may be responsible for its anti‐OSCC effects.

The importance of vitamin D in cancer management has been previously investigated (Anand et al., 2017; Bochen et al., 2018). In patients with advanced cancers, it has been shown that vitamin supplementation reduced therapy‐related toxicities and improved the overall quality of life of the patients (Anand et al., 2017; Bochen et al., 2018).

In the present study, we focused on the estimation of serum vitamin D levels of OSCC patients in a Middle Eastern subpopulation and made comparisons with cancer‐free controls. This is intended to show any associations between serum vitamin D level and the development of OSCC. More than 74% of the OSCC patients from our study had moderate to severe deficiency of vitamin D. This is in keeping with what was previously reported by other authors (Anand et al., 2017; Bochen et al., 2018 and Grimm et al., 2015). Grimm et al. (2015) reported that among the 42 OSCC patients in their study in a Caucasian population, 100% of them had moderate to severe vitamin D deficiency, while Anand et al. (2017) reported 76.4% deficiency among the tested group. Bochen et al. (2018) in an analysis of 231 patients with HNSCC reported a significantly lower median serum vitamin D levels among the cancer cases when compared to the cancer‐free controls.

This very high prevalence of vitamin D deficiency among oral cancer patients, generally, therefore raises the question as to the significance of this nutrient in the etiopathogenesis of OSCC and by implication its place in the prevention of the disease. It is important to note that a significant number of OSCC patients in our study have moderate to severe deficiencies of vitamin D in comparison to the cancer‐free controls. Overall, the estimated mean serum vitamin D levels were also significantly lower for the cancer group than the controls. Logistic regression analysis shows a positive association between vitamin D deficiency and OSCC risk, especially in levels below 25 ng/ml with an OR of 1.65. This means that subjects with moderate to severe vitamin D deficiency from the present study have a 1.65 increase in odds of developing OSCC.

Epidemiological studies in Saudi Arabia, the location of our study, show that vitamin D deficiency is prevalent in the general population (Al‐Alyani, Al‐Turki, Al‐Essa, Alani, & Sadat‐Ali, 2018; Alsuwadia et al., 2013). This makes the findings of this study even more significant and relevant to this population.

## 5 CONCLUSION

The results of the present study further corroborate the assertion that vitamin D deficiency may be a useful indicator or pointer of OSCC risk. It may, therefore, be necessary to routinely assess vitamin D status of patients attending our medical and dental outpatient clinics and to prescribe vitamin D supplements to subjects with moderate to severe deficiencies in order to decrease the chances of OSCC development. However, further multicenter study involving a larger sample size would be necessary to confirm the results of this study in this part of the world.
